# Anastomotic Rings and Inflammation Values as Biomarkers for Leakage of Stapled Circular Colorectal Anastomoses

**DOI:** 10.3390/diagnostics12122902

**Published:** 2022-11-22

**Authors:** Feng Zhang, Song Qiao, Ning Yao, Chunqiao Li, Marie-Christin Weber, Benedict Jefferies, Helmut Friess, Stefan Reischl, Philipp-Alexander Neumann

**Affiliations:** 1 Department of Surgery, Klinikum rechts der Isar, School of Medicine, Technical University of Munich (TUM), 81675 Munich, Germany; 2 Department of General Surgery, Tongren Municipal People’s Hospital of Guizhou Medical University (GMU), Tongren 554300, China; 3 Department of Pathology, Tongren Municipal People’s Hospital of Guizhou Medical University (GMU), Tongren 554300, China; 4 Institute of Diagnostic and Interventional Radiology, Klinikum rechts der Isar, School of Medicine, Technical University of Munich (TUM), 81675 Munich, Germany

**Keywords:** anastomotic ring, colorectal anastomosis, anastomotic leakage, neutrophil to lymphocyte ratio, stapler anastomosis

## Abstract

Reliable markers to predict or diagnose anastomotic leakage (AL) of stapled circular anastomoses following colorectal resections are an important clinical need. Here, we aim to quantitatively investigate the morphology of anastomotic rings as an early available prognostic marker for AL and compare them to established inflammatory markers. We perform a prospective single-center cohort study, including patients undergoing stapled circular anastomosis between August 2020 and August 2021. The predictive value of the anastomotic ring configuration and the neutrophil-to-lymphocyte ratio (NLR) regarding anastomotic leakage is examined by ROC analyses and compared to the C-reactive protein (CRP) as an established marker. We included 204 patients, of which 19 suffered from anastomotic leakage (LEAK group), while in 185 patients the anastomoses healed well (HEAL group). The minimal height of the anastomotic rings as a binary classifier had a good ROC-AUC of 0.81 but was inferior to the NLR at postoperative day (POD) 5, with an excellent ROC-AUC of 0.93. Still, it was superior to the NLR at POD 3 (0.74) and the CRP at POD 3 (ROC-AUC 0.54) and 5 (ROC-AUC 0.70). The minimal height of the anastomotic rings as indicator for technically insufficient anastomoses is a good predictor of AL, while postoperatively the NLR was superior to the CRP in prediction of AL.

## 1. Introduction

Anastomotic leakage (AL) is currently the most common serious complication after colorectal surgery [1,2]. Although the technical skills have greatly improved in recent years, AL can still be detected in up to 19% of cases [3,4]. The occurrence of postoperative AL in surgery for colorectal cancer (CRC) increases the mortality of patients and may also lead to a higher local recurrence rate and a drop of long-term survival rate [5,6]. Therefore, in order to effectively improve the tumor-specific survival, early detection and timely treatment of AL following colorectal surgery is of great importance.

Although patients with AL often present with non-specific clinical symptoms such as a fever, leukocytosis, dyspnea or abdominal distension, these symptoms often occur after the manifestation of AL and cannot be used for prediction of AL after colorectal surgery [3,7]. Therefore, the use of serum inflammatory markers to detect AL early on is established, among which the C-reactive protein (CRP) is most commonly used [8]. From the fourth day after surgery, CRP levels can be used as reliable indicators for AL [9].

However, heterogeneous patient cohorts in previous studies, as well as inconsistent diagnostic criteria for AL and cut-off values of postoperative CRP have affected the application value of CRP as an early predictor of AL. Therefore, finding reliable and easily detectable predictors in the prevention of AL still has a high clinical significance.

In recent years, intraluminal stapling techniques have been widely used, especially in laparoscopic surgery [10]. So far, anastomotic rings have not received considerable attention as potential risk factor for AL and exact data about the differences in ring morphology is lacking. Their integrity is usually examined qualitatively, and the stapler manufacturers do not define the target size range of the rings. As mentioned above, established laboratory makers for AL such as CRP already have a certain value in predicting postoperative AL [11]. Another indicator to predict AL, the neutrophil-to-lymphocyte-ratio (NLR), has been established in recent years [12,13]. However, both inflammatory markers have the limitation of being available postoperatively, while the ring measurements are already available intraoperatively and could have an important impact by triggering early countermeasures to avoid the development of AL.

In this study, we aim to investigate the morphological configuration of anastomotic rings as an early available prognostic marker for AL and compare the prognostic value to the NLR and CRP as classifiers of AL occurrence.

## 2. Materials and Methods

### 2.1. Patient Collective

This study was approved by the Ethics Committee of Tongren Municipal People’s Hospital of Guizhou Medical University [(2020)47-4] at 31 July 2020. Informed consent was obtained from all patients included in the study. Patients were included during the study period from 10 August 2020 to 12 August 2021. Patient data were collected during the study period, including gender, age, ASA score, Body Mass Index (BMI), operation time, intraoperative blood loss, CRP and NLR at the Tongren Municipal People’s Hospital of Guizhou Medical University.

### 2.2. Inclusion and Exclusion Criteria

Patients undergoing surgical resection of pathologically confirmed CRC with subsequent stapled circular anastomosis were included in the study. Patients with the following confounders were excluded: preoperative signs of infection, concomitant hematological diseases, additional diagnoses of malignant diseases, preoperative radio-/chemotherapy or preventive ileostomy.

### 2.3. Configuration of Anastomotic Rings

**Figure 1:**
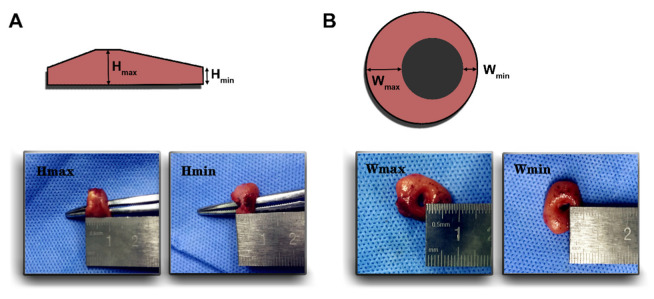
**Measurement of anastomotic ring configuration.** (**A**) Minimal height (H_min_) and maximal height (H_max_), (**B**) minimal width (W_min_) and maximal width (W_max_), were measured intraoperatively.

To measure the configuration of anastomotic rings during the operation, the surgeons first observed the integrity of the rings, and then using a sterile ruler to measure the minimal height (H_min_), maximal height (H_max_), minimal width (W_min_) and maximal width (W_max_) of the fresh rings (Figure 1A,B). The measurements were performed by two surgeons independently. In case of inconsistent results, consent was achieved by repeated measurement of the surgeons together.

### 2.4. Diagnostic Criteria of Anastomotic Leakage

During the inpatient treatment time until the first follow-up 14 days postoperatively, AL was diagnosed if one or more of the following occurred in the period of the hospitalization and postoperative follow-up: (1) Proof of anastomotic defect by digital rectal exam, colonoscopy or rectoscopy; (2) Purulent or stool-like secretion of the pelvic drainage; (3) Radiologically confirmed perianastomotic abscess formation or anastomotic defect; (4) Rectovaginal or rectovesicular fistula.

### 2.5. Statistical Methods

SPSS 28.0 (IBM, USA) software was used for statistical analyses. Ratio data were presented as mean ± standard deviation (mean ± SD) in tables and box plots in diagrams, categorical data were presented as frequencies (in %). Chi-square test was used for the comparison of categorical data. Two-sided unpaired T-test was used for the comparison of ratio scaled, Gaussian distributed data. Receiver Operating Characteristics (ROC) as well as Area-Under-the-Curve (AUC) analyses were used to evaluate the ring measures and the NLR and the CRP as binary classifier to predict AL postoperatively. Optimized cut-off values were determined by maximizing the Youden index. Values of *p* < 0.05 were considered statistically significant.

## 3. Results

### 3.1. Patients’ Clinical Characteristics

In total, 247 patients underwent resection for colorectal cancer followed by circular stapler anastomosis in Tongren Municipal People’s Hospital of Guizhou Medical University during the period from 10 August 2020 to 12 August 2021. Among these 247 patients, 43 cases were excluded for meeting the exclusion criteria. The resulting 204 patients had a mean age of 63.2 ± 11.6 years and a mean BMI of 22.8 ± 2.1. The collective consisted of 83 females and 121 males. The patients included were staged for stage II or III according to the UICC/AJCC classification (8th Edition) and underwent an anterior resection of the proximal rectum and the distal sigmoid colon with a sigmoidorectostomy. All operations were performed by a single surgeon (S. Q.) and were completed laparoscopically with his surgical group. All procedures were carried out without conversion to conventional laparotomy. The specimens were removed through left lower quadrant incision. The mean operation time was 161.4 ± 31.1 min and the mean intraoperative blood loss was 175.8 ± 38.1 mL (Table 1).

### 3.2. Configuration of Anastomotic Rings

To perform the anastomosis, two different stapler sizes were used: CDH25A and CDH29A (Ethicon Endo-Surgery LLC, USA). Although the diameters of the rings differed between the stapler types, there were no statistical differences between the two stapler types regarding all other measurements of anastomotic rings (Table 2).

The measurement results of the rings in 204 patients were as follows: the ring H_min_ value was statistically lower in the LEAK group than that of the HEAL group (4.4 ± 0.5 mm vs. 5.2 ± 0.8 mm; *p* < 0.001). H_max_, W_min_ and W_max_ did not show statistical differences between the LEAK group and the HEAL group (Table 1).

### 3.3. H_min_ as Binary Classifier to Predict Anastomotic Leakage

Anastomotic leakage occurred in 19 of 204 patients (9.3%) after a mean of 5.8 ± 1.4 days. Compared with the 185 cases in HEAL group, there was no statistical difference in terms of gender, age, ASA score, BMI, operating time, intraoperative blood loss and tumor stage (Table 1).

**Figure 2:**
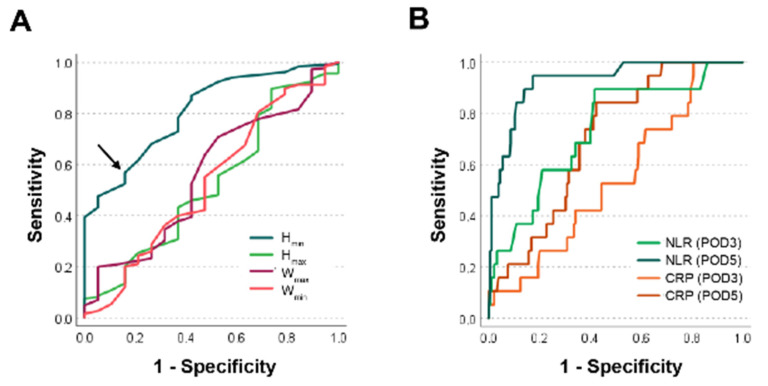
**Receiver operating characteristic analyses for ring measurements and the inflammatory markers.** (**A**) Receiver operating characteristic (ROC) curve for minimal and maximal height and minimal and maximal width of anastomotic rings to predict anastomotic healing are shown. The Youden index-optimized cut-off value for the best predictor H_min_ at 4.95 mm is indicated by an arrow. (**B**) ROC-analyses of the postoperative NLR and the CRP at postoperative day (POD) 3 and 5 are shown.

To further analyze H_min_ as a potential binary classifier for anastomotic leakage, the optimal cut-off value of 4.95 mm was determined by maximizing the Youden index. At this value, the sensitivity for predicting AL following colorectal surgery was 56.8% with a specificity of 84.2% (Figure 2A).

Of the 204 cases, 108 cases had minimal anastomotic ring heights of more than 4.95 mm, of which three (2.8%) developed AL. In 96 cases, minimal anastomotic ring heights were below 4.95 mm, of which 16 (16.7%) developed AL. Between the two groups separated by an H_min_ value of 4.95 mm, the difference in leakage rate was statistically significant (χ^2^ = 11.6, *p* < 0.001), as shown in Table 3. Other factors such as gender, age, ASA score, BMI, operating time, intraoperative blood loss and tumor stage did not show any statistical difference between the former (H_min_ above 4.95 mm) and the latter group (H_min_ below 4.95 mm) (Table 4).

### 3.4. Postoperative Development of NLR and CRP Values

**Figure 3:**
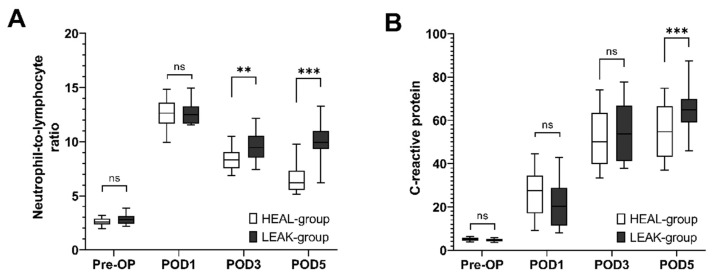
**Time course of the neutrophil-to-lymphocyte ratio (NLR) and C-reactive protein (CRP) values perioperatively**. (**A**) NLR values preoperatively and at postoperative day (POD) 1, 3 and 5 are shown between the groups. (**B**) CRP values are shown for the same points in time as in (**A**). Median values (bar), 25–75% quartile (box) and 5–95% quartile (whiskers) are shown. Statistical differences determined by unpaired t-test are indicated with ** (*p* < 0.01), *** (*p* < 0.001).

In order to compare H_min_ to established inflammatory markers, the NLR and the CRP values were monitored and analyzed perioperatively. Compared to the preoperative measurements (2.8 ± 0.5), the NLR markedly increased at POD 1 (12.7 ± 1.0), and then stabilized (POD 3: 9.5 ± 1.3; POD 5: 10.0 ± 1.6) in the LEAK group. Patients in the HEAL group had NLR values of 2.6 ± 0.4 preoperatively. A postoperative increase to 12.6 ± 1.4 was followed by a gradual decrease to 8.4 ± 1.1 and 6.7 ± 1.5 postoperatively. The values between the groups were statistically different at POD 3 (*p* < 0.002) and POD 5 (*p* < 0.001) (Figure 3A).

Compared to preoperative values (4.7 ± 0.7 mg/L), the CRP kept increasing postoperatively in the LEAK group (POD 1: 22.3 ± 11.2 mg/L; POD 3: 53.8 ± 13.0 mg/L; POD 5: 64.9 ± 10.4 mg/L). Patients in the HEAL group had CRP values of 5.1 ± 0.8 mg/L, followed by a less pronounced postoperative increase of 26.3 ± 11.0 mg/L, 52.0 ± 13.3 mg/L, 55.2 ± 12.6 mg/L. The values between the groups were statistically different at POD 5 (*p* < 0.001) (Figure 3B).

### 3.5. Accuracy of H_min_ Compared to NLR and CRP as Binary Classifiers for Anastomotic Leakage

The ROC curve showed only a moderate ROC-AUC of 0.74 for NLR at POD 3, while it was even poorer at 0.54 for CRP at POD 3. For CRP at POD 5 the ROC-AUC of 0.70 was also only moderate. The NLR had very good ROC-AUC of 0.93 at POD 5 (Figure 2B) (Table 5). H_min_ outperformed all values except the NLR at POD 5 with a good ROC-AUC value of 0.81 (Figure 2A) (Table 5).

## 4. Discussion

Intraluminal stapling techniques for colorectal anastomoses have been widely accepted nowadays and are a standard choice for reconstruction following colorectal surgery, particularly suitable for those who are undergoing laparoscopic operations [10]. Anastomotic rings are retrieved during the formation of colorectal anastomoses with circular staplers. This study shows that the anastomotic ring configuration, especially for H_min_, is critical for the risk of suffering postoperative leakage, as H_min_ is a significant predictor for development of AL in this prospective study, a result that was similarly reported by one other group [14]. Above the optimal decision point of 4.95 mm, determined by the Youden index, the leakage rate is significantly lower than that below that point. Additionally, we could show that, in the postoperative period, the NLR is superior to the CRP in diagnosis of anastomotic leakage.

In this study, H_min_ with a ROC-AUC of 0.81 has a superior predictive performance to the NLR at POD 3 in predicting AL with a ROC-AUC of 0.74. Still, it is inferior to the NLR at POD 5, with an outstanding ROC-AUC of 0.93. Considering the average time point for the occurrence of leakage of 5.8 days postoperatively, the NLR at POD 3 or 5 appears suitable to diagnose but not to prevent the development of AL by appropriate countermeasures. As shown above, the ring configuration is a significant real-time predictor for AL. Therefore, H_min_ is a useful tool to identify technical problems of anastomosis formation allowing the decision for immediate intraoperative revision of the anastomosis.

Additionally, our study has confirmed that compared to CRP, the NLR as a leakage marker is not only simpler and available at a lower cost, but has better efficiency as well [15]. In our study, the NLR was already significantly different between the HEAL group and the LEAK group at POD 3 compared to the CRP, which did not differ at POD 3 but only at POD 5 between the groups. Other studies have shown that the NLR can be used to predict perioperative complications in patients undergoing colorectal surgery [16,17]. A study of Cook et al. shows that for patients undergoing colorectal resections, at a NLR ≥ 9.3 on the third day after surgery, complications are more likely to occur [18]. The NLR can also reflect the severity of systemic inflammation, the study of Mik et al. confirms that the NLR of patients dying from AL on the fourth day after operation is higher than that of other patients with AL [19].

Although being the largest prospective single center cohort study in this field, this study has some limitations that should be discussed. An important limitation of our study is the limited number of included patients. To validate our findings further, additional (multi-center) studies will be needed. The loss to follow-up cannot be excluded for patients suffering from late AL after being discharged from the hospital. Still, the risk should be low, as AL usually occurs early and within the postoperative in-house treatment. Although gender, age, ASA score, BMI, operation time, blood loss and tumor stage did not differ between the groups, we cannot exclude other confounders to affect the occurrence of AL in our analysis.

## 5. Conclusions

In summary, our study confirms that proper configuration of the anastomotic rings is essential to prevent leakage. A low minimal height represents a technical flaw and can indicate the risk to develop leakage with a high specificity, and thus should be evaluated intraoperatively. In the postoperative period, the NLR is superior to the established CRP to identify AL and should be preferred.

## Figures and Tables

**Table 1:** Patient characteristics and the configurations of anastomotic rings.

Items	Total (n = 204)	Groups		*p*-Value
		LEAK (n = 19)	HEAL (n = 185)	
Gender (m/f)	121/83	12/7	109/76	*0.72*
Age	63.2 ± 11.6	66.2 ± 11.8	62.9 ± 11.5	*0.24*
ASA Score	2.2 ± 0.4	2.1 ± 0.3	2.2 ± 0.4	*0.24*
BMI (kg/m^2^)	22.8 ± 2.1	22.5 ± 1.9	22.9 ± 2.1	*0.46*
OP time (min)	161.4 ± 31.1	161.1 ± 33.5	161.4 ± 30.9	*0.96*
Blood loss (mL)	175.8 ± 38.1	165.8 ± 44.5	176.8 ± 37.3	*0.23*
UICC/AJCC stage (II/III)	143/61	13/6	130/55	*0.87*
Distance to anal verge (>10 cm/<10 cm)	114/90	10/9	104/81	*0.76*
H_min_ (mm)	5.1 ± 0.8	4.42 ± 0.45	5.21 ± 0.78	*<0.001*
H_max_ (mm)	8.7 ± 1.0	8.57 ± 1.03	8.71 ± 1.03	*0.58*
W_min_ (mm)	4.9 ± 0.7	4.86 ± 0.78	4.94 ± 0.72	*0.68*
W_max_ (mm)	8.6 ± 0.9	8.46 ± 0.81	8.65 ± 0.90	*0.37*
NLR preop	2.6 ± 0.4	2.8 ± 0.5	2.6 ± 0.4	*0.10*
NLR at POD1	12.6 ± 1.4	12.7 ± 1.0	12.5 ± 1.4	*0.69*
NLR at POD3	8.5 ± 1.2	9.54 ± 1.31	8.44 ± 1.12	*0.002*
NLR at POD5	7.0 ± 1.8	10.03 ± 1.55	6.65 ± 1.45	*<0.001*
CRP preop (mg/L)	5.0 ± 0.8	4.7 ± 0.7	5.0 ± 0.8	*0.06*
CRP at POD1 (mg/L)	26.0 ± 11.0	22.3 ± 11.2	26.3 ± 11.0	*0.15*
CRP at POD3 (mg/L)	52.1 ± 13.3	53.7 ± 13.0	52.0 ± 13.3	*0.57*
CRP at POD5 (mg/L)	56.0 ± 12.7	64.90 ± 10.43	55.17 ± 12.61	*<0.001*

H_min_ = minimal height of the ring; H_max_ = maximal height of the ring; W_min_ = minimal width of the ring; W_max_ = maximal width of the ring; NLR = Neutrophil-to-lymphocyte ratio, CRP = C-reactive protein; POD = postoperative day.

**Table 2:** Anastomotic ring configuration of different stapler types.

Items	Mean ± SD (in mm)		*p*-Value
	CDH25A (n = 69)	CDH29A (n = 135)	
H_min_	5.21 ± 0.82	5.09 ± 0.77	*0.30*
H_max_	8.76 ± 0.96	8.66 ± 1.07	*0.50*
W_min_	4.99 ± 0.77	4.90 ± 0.70	*0.37*
W_max_	8.73 ± 1.04	8.59 ± 0.80	*0.28*

H_min_ = minimal height of the ring; H_max_ = maximal height of the ring; W_min_ = minimal width of the ring; W_max_ = maximal width of the ring.

**Table 3:** Anastomotic leakage rate in subgroups separated by the optimal H_min_ cut-off of 4.95 mm.

Cut-Off	Total (n)	Groups		Leakage Rate
		LEAK (n)	HEAL (n)	
>4.95 mm	108	3	105	2.8% ^1^
<4.95 mm	96	16	80	16.7% ^1^
Total	204	19	185	9.3%

^1^ Chi-square value χ^2^ = 11.6; *p* < 0.001.

**Table 4:** Comparison of the clinical and demographical data between the subgroups separated by H_min_.

Items	Total (n = 204)	Cut-Off Value (4.95 mm)		*p*-Value
		Above (n = 108)	Below (n = 96)	
Gender (m/f)	121/83	63/45	58/38	*0.76*
Age	63.2 ± 11.6	62.5 ± 11.6	63.96 ± 11.5	*0.38*
ASA Score	2.2 ± 0.4	2.2 ± 0.4	2.2 ± 0.4	*0.79*
BMI (kg/m^2^)	22.8 ± 2.1	22.9 ± 2.1	22.8 ± 2.1	*0.74*
OP time (min)	161.4 ± 31.1	161.0 ± 33.0	161.8 ± 29.0	*0.86*
Blood loss (mL)	175.8 ± 38.1	178.9 ± 37.4	172.3 ± 38.7	*0.22*
UICC Stage (II/III)	143/61	79/29	64/32	*0.31*
Distance to anal verge (> 10 cm/< 10 cm)	114/90	64/44	50/46	*0.30*

**Table 5:** ROC-AUC values of the different classifiers.

Classifier	ROC-AUC
H_min_	0.81
H_max_	0.53
W_min_	0.53
W_max_	0.56
NLR at POD3	0.74
NLR at POD5	0.93
CRP at POD3	0.54
CRP at POD5	0.70

## Data Availability

Patient data were retrieved from Tongren Municipal People’s Hospital of Guizhou Medical University. The data that support the findings of this study are not publicly available due to their containing information that could compromise the privacy of research participants, but are available from the first author Feng Zhang and the second author Song Qiao upon reasonable request.
